# A Study on Secure Medical-Contents Strategies with DRM Based on Cloud Computing

**DOI:** 10.1155/2018/6410180

**Published:** 2018-03-29

**Authors:** Hoon Ko, Libor Měsíček, Jongsun Choi, Seogchan Hwang

**Affiliations:** ^1^IT Research Institute, Chosun University, 309 Pilmun-daero, Dong-gu, Gwangju 61452, Republic of Korea; ^2^Jan Evangelista Purkyně University in Ústí nad Labem, Pasteurova 1, 400 96 Ústí nad Labem, Czech Republic; ^3^School of Computer Science and Engineering, Soongsil University, 369 Sangdo-Ro, Dongjak-Gu, Seoul 06978, Republic of Korea; ^4^Gensoloft Inc., 99 Jangseungbaegi-Ro, Dongjak-Gu, Seoul 06936, Republic of Korea

## Abstract

Many hospitals and medical clinics have been using a wearable sensor in its health care system because the wearable sensor, which is able to measure the patients' biometric information, has been developed to analyze their patients remotely. The measured information is saved to a server in a medical center, and the server keeps the medical information, which also involves personal information, on a cloud system. The server and network devices are used by connecting each other, and sensitive medical records are dealt with remotely. However, these days, the attackers, who try to attack the server or the network systems, are increasing. In addition, the server and the network system have a weak protection and security policy against the attackers. In this paper, it is suggested that security compliance of medical contents should be followed to improve the level of security. As a result, the medical contents are kept safely.

## 1. Introduction

A health care system needs some network devices such as smart devices, servers, and sensors based on a network, where the server is storing all of the patients' medical information. The information in the system is used by a doctor or medical experts to monitor all of the patients' medical status remotely on a network. To connect to them, they can use Bluetooth or WiFi and other network technologies that can be used in health care systems. However, because of the systems process in the network, security problems such as cyberattacks certainly can appear in the system [1]. The damage will be serious; for example, in case there is a security accident like patient medical information leakage that contains a patient's disease name and all medical records, it can lead to a privacy problem. In addition, the health care system uses a large amount of data with records about the patients' disease to predict potential medical attacks or a sudden critical status. When it decides to analyze a small data set, the health care system's large data technology collects the pattern of disease flow, which would come from heterogeneous devices. Next, with the analyzed results, a medical system or a doctor observes each patient's status. Then each prescription is suggested automatically, and these records are saved in a server in the health care system. As it was said, when this is used, the system may have a privacy problem due to cyberattacks. To protect the records, all users need to log in their ID/password with an encryption algorithm. As well, the encryption algorithm is used when the patents' records are saved [2]. Now, the network that the system uses, and that which it has been using, has not enough protection against cyberattackers with just an ID/password [3]. Even if it has a security program, the security policy has to be updated periodically or automatically with security processes such as DRM agent or server, Key Management Policy, License Policy, and Security Policy. However, more important is how to keep the medical records following particular rules, which should consider each medical record. Therefore, this paper suggests the security management of medical records in a health care system on the cloud [4] and shows its safety. Security management, as its name suggests, contains a DRM server, DRM agent, License Policy, Security Policy, and Key Management Policy. With these components, it is able to manage all security rules and policies in order to reduce the potential and impact of risks from attacks, including cyberattacks, that can be discovered in advance [1]. The rest of the paper is divided as follows: Section 2 describes related works, Section 3 explains safe medical record management, Section 4 contains the analysis, and Section 5 presents the conclusions.

## 2. The Related Works

### 2.1. Medical Records

**Figure 1:**
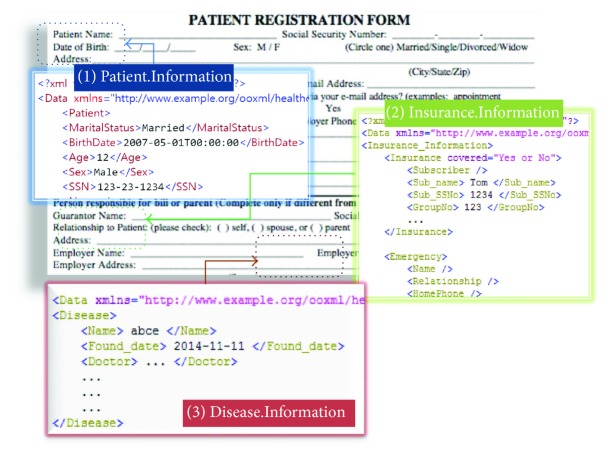
Patient registration form.

Microsoft provides usage with an Office Open XML format which supports portable medical electronics, a standard within the medical industry with Office 2007 [5, 6]. Also, many relevant institutes have been working with this similar area. As shown in Figure 1, Hoon Ko had defined the medical record form that normally contains 3 categories: *(1) Patient.Information* which consists of name, birth information, and address; *(2) Insurance.Information* which includes the insurance information of the patient and a phone number in case of an emergency; and *(3) Disease.Information* that involves detailed disease information [7]. Almost all of the health care system, which consists of network elements, contains private and sensitive information [8]. While it collects the data from various sources, the information exposure will be increased. Then the attacker tries to use the exposed information to make useful information. To use the system safely, users are usually operating with a current key-management system to encrypt. However, it is a classic model that is being applied and it is difficult to apply into a new health care system [2]. M. B. Jain et al. and Khan and Zhang had studied user interface requirements for web-based integrated care pathways [9, 10]. The aim is to address this gap by evaluating the usability of a novel web-based tool called COCPIT (Collaborative Online Care Pathway Investigation Tool), which supports the design, analysis, and visualization of ICT at the population level. Patients and doctors use this interface to analyze their state by connecting to a server. Now this idea is planned to be used only in a local area, like in a hospital. As viewed in [11], Amin et al. had presented a user authentication scheme for Telecare Medical Information System (TMIS), which can use the Internet between a patient and a medical server [12–14]. To overcome the security weakness, they had designed a medical system architecture and a standard mutual authentication scheme to exchange medical data. Also through this scheme, they share the key [5, 9, 10]. They had used elliptic curve cryptography which is a good algorithm in a mobile device [15].

### 2.2. Threats and Damages

This section contains the threats to the medical contents. Security and medical record protection issues are of importance in the adoption of a cloud-based health care solution. We have summarized cyberattack and damage records in Tables 1 and 2.

### 2.3. DRM Storage System

**Figure 2:**
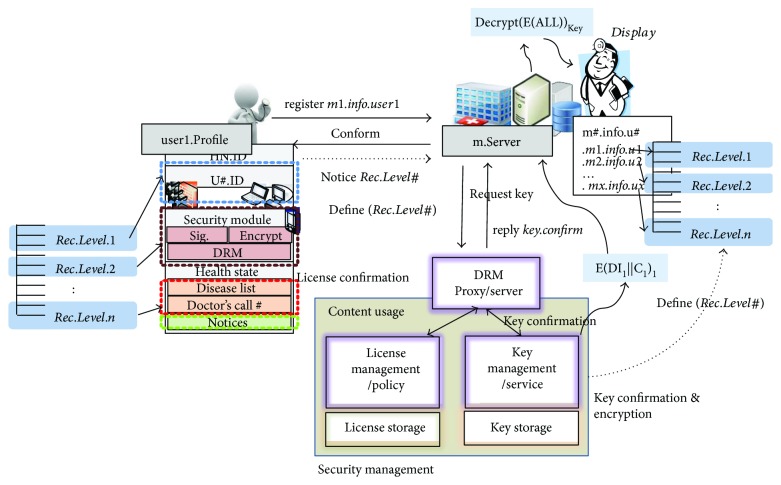
Security process with DRM.

The DRM storage system runs independently of the health care system. This system includes DRM register for registration, DRM test module for testing DRM consistency, DRM interoperable module for delivering DRM metadata, and DRM information module for sharing DRM registration, sales, and usage information with respective media service systems [16]. Security management in Figure 2 shows a structure of the DRM storage and service system. It does not manage DRM metadata in the system or the media service system because a DRM can be freely registered or released by DSP. This system retrieves DRM-related information from the DRM server provided by the DSP and provides the information to the media service system when it is necessary.

## 3. Safe Medical Record Management

### 3.1. Medical Records with DRM

Figure 2 shows the suggested security process with DRM. There are three components in medical record management with DRM: user#.Profile, m.Server, and DRM server. The user#.Profile contains *Patient.Information*, *Insurance.Information*, and *Disease.Information*. The m.Server keeps user*m#.info.u#* that has all patients' medical records like *m1.info.u1*, *m2.info.u2*, *…* , *mx.info.ux*. The DRM consists of License Policy Service and Key Management Service which communicates to m.Server in m#.info.u# [17].

There are two security issues, signature/encryption and security level and lightweight cryptography in [18, 19]. For *signature and encryption*, it takes the size of an input key and the number of encryption rounds after analyzing a new structure that the computer plans to create when the physician in charge takes their patients' medical records following their disease level and the patients' status (Table 3). *N*_B_ is the number of 32-bit words depending on the size of encryption block, *N*_K_ is the number of 32-bit words depending on the key length of an encryption, and then *N*_R_ which is the number of rounds is *N*_R_ = 6 + max (*N*_B_, *N*_K_). The length of the AES block is 128 bits and because it supports such 4 bits to NB, 128 bits, 192 bits, and 256 bits to AES, therefore *N*_K_ gets 4, 6, and 8. Finally, the value in each round to each bit will be *N*_R_ = 6 + max (*N*_B_, *N*_B_) = 6 + max ([4, 128], [6, 196], [8, 256]) = (10, 12, 14). *Lightweight cryptography* is a cryptographic algorithm for implementation in constrained environments including a sensor and a smart card in a health care system. It consists of a hardware implementation and a software implementation. In the hardware implementation case, physical size and energy consumption are very important to decide as to how much should be spent. On the other hand, in the software implementation case, smaller code and lesser memory size are suitable in lightweight cryptography. The following items are the reasons why lightweight cryptographic algorithms are required [18, 19].

### 3.2. DRM System Module

The DRM interoperable module manages metadata by using information from the DRM server and exchanges the information with the DRM module in a system. It uses a DIF (DRM interoperable format) document, which extends CPIX (Content Protection Information Exchange format) technology for exchanging the content protection information and DRM metadata information. The DRM consistency test module checks whether the DRM metadata information is correctly received for the registered DRM and whether it can be used in the service. The pregenerated DIF v1.0 document (if updated to v2.0 and v3.0 through the DRM interoperable module and external DRM service, resp.) and the contents of the final document are examined and judged. The DIF v3.0 document should contain accurate DRM metadata information and information about authentication and decryption for CP.

### 3.3. Secure Medical Contents with DRM

The suggested electronic registration form (ERF) consists of *ERF = [(1) Patient.Info(PI) *||* (2) Insurance.Info(II) *||* (3) Disease.Info(DI)]_n_]*, and each will be stored independently. It only takes the records that it wants and needs. And because a patient could have multiple diseases, (3) DI can be acceptable for multiple storing. PI links to II and to (PI_*n*_ → II_*n*_) and also to DI and to (PI_*n*_ → DI_*n*_); on the other hand, there is nothing to link between II and DI. It means that only PI can call what disease records it exactly needs which is stored after encrypting, because PI is linked to each table of DI. Next, when the server is asked to perform, all it has to do is decrypt only the data which is requested. It effects the reduction of cost such as (*n*∗*t*) → (*n*/*i*)∗*t* as it has to decrypt all diseases [18].

## 4. Analysis

### 4.1. Security Strategies

In Section 4.3, it is suggested that a health care system with security contains medical record security and channel security between a patient and a server in a hospital. There are seven threats such as repudiation, tampering, spoofing, DDoS, information disclosure, eavesdropping/forgery modulation attack to medical record, and exposure of personal information by medical information sharing.

### 4.2. Security Level Decision

ERF is structured for the records to be stored regarding each purpose of the records. It can be possible to partially encrypt only what it needs. There are two ways to decide on the security level. To define strong security strength, the first way is to store after encrypting the entire records such as disease name and its symptom; its cost will be *E*(DI||*C*). On the other hand, the second way is to store after encrypting only the symptom; it does not encrypt the disease name. In the two cases, the strength costs expects next; the cost of the first way is 1 − [DI/(DI + *C*)]∗100∗*T*, and the second cost will be [1 − [*C*/(DI + *C*)]∗100]∗*T*.

### 4.3. Threats to Model and Solutions

#### 4.3.1. Repudiation

It contains lower trusted subject update logs, data logs from an unknown source, insufficient auditing, data storage denying a device from potentially writing data, and potential data repudiation by a server. Repudiation threats involve an adversary denying that something happened.

- Lower trusted subject update logs: letting everyone write to your logs can lead to repudiation problems.
- Data logs from an unknown source: it involves an adversary denying that something happened.
- Insufficient auditing: you might want to talk to an audit expert as well as a privacy expert about your choice data.
- Data store denies device potentially writing data: patients claim that they did not write the data received from an entity on the other side of the trust boundary.
- Potential data repudiation by a server: medical server claims that it did not receive data from a source outside the trust boundary.

**Solution 1 .**

To protect the patients' medical records, sensors have to confirm their unique number. Role_sensor_ ← (u#.profile||u#.devices) Repudiation to device and stability of sensor (patients' number identify) and the patients' information stored in the sensor is formulated → (flow description || safety symbol).

#### 4.3.2. Tampering

It contains replay attacks, collision attacks, and risks from logging. The device data store could be corrupted and authenticated dataflow compromised. It is the act of altering the bits. Tampering with a process involves changing bits in the running process. Similarly, tampering with a dataflow involves changing bits on the wire or between two running processes.

- Replay attacks: packets or messages without sequence numbers or time-stamps can be captured and replayed in a wide variety of ways.
- Collision attacks: attackers who can send a series of packets or messages may be able to overlap data.
- Risks from logging: log readers can come under attack via log files.
- Possible corruption of the data storage device: data flowing across generic dataflow may be tampered with by an attacker.
- Authenticated dataflow compromised: an attacker can read or modify data transmitted over an authenticated dataflow.

**Solution 2 .**

To solve existing problem, integration processing has to be set to all medical records in a server and in a device. And only an authorized person can modify or edit the medical records. Following their authorization level, the person only can take a look at the record which is on the same authorization level.

#### 4.3.3. Spoofing

There is a destination data storage security manager, a source data storage device in spoofing, and it is when a process or entity is something other than its claimed identity. Examples include substituting a process, a file, website, or a network address.

- Destination data storage security manager: the security manager may be spoofed by an attacker, and this may lead to data being written to the attacker's target instead of the security manager.
- Source data storage device: the patient may be spoofed by an attacker, and this may lead to incorrect data delivered to the medical server.

**Solution 3 .**

The system has to check if the medical records are right from a patient, a server, and a DRM. Also, although the attacker intercepts the data, they cannot read the medical records without a security key by using a security module such as encryption.

#### 4.3.4. DDoS

It consists of potential excessive resource consumption for DRM, data storage inaccessibility, and dataflow—generic dataflow is potentially interrupted and resource consumption attacks can be hard to deal with, and there are times that it makes sense to let the OS do the job.

- Potential excessive resource consumption for DRM: denial of service happens when the process or data storage is not able to service incoming requests or perform up to spec.
- Data storage inaccessibility: an external agent prevents access to a data storage on the other side of the trust boundary.
- Dataflow—generic dataflow is potentially interrupted: an external agent interrupts data flowing across a trust boundary in either direction.

**Solution 4 .**

To solve them, it is necessary to authenticate, which is by a new authentication method, not with a simple ID/PW method but by ID card, used for legal access by a patient or a responsible doctor. And also, it has to be taken into account that a special key is used which encrypts and decrypts the unique doctor's ID information. The server in the hospital or medical center has to control the unique ID information.

#### 4.3.5. Information Disclosure

Weak authentication scheme, authorization bypass, weak credential storage, and weak access control for a resource belong to information disclosure. This happens when it can be read by an unauthorized party.

- Weak authentication scheme: custom authentication schemes are susceptible to common weakness.
- Authorization bypass: it can access a security manager and bypass the permission for the object.
- Weak credential storage: credentials held at the server are often disclosed or tampered with and credentials stored on the client are often stolen.
- Weak access control for a resource: improper data protection of patient lists can allow an attacker to read information not intended for disclosure.

**Solution 5 .**

The medical center has to ask all of the staff to follow the center's security policies by updating authentication policies periodically such as restriction of information use, deleting, and copying. Next, output control of patients' medical records is needed, keeping the log files when they access their records.

## 5. Conclusion

This studied the security level of medical records, which contains patients' personal information, patient insurance information, and patients' diseases list following the number of disease. Usually, a medical system gets the bioinformation by using sensors for biometrics. An addition to this software implementation could change this procedure and its possible impact to this algorithm. For example, automatic identification and data capture (AIDC) technology, such as sensors for iris, facial, fingerprint, or vocal recognition could allow and record biometric data which are unique to each individual. This reason could enhance the security of algorithm-based strategies. The medical record size from the health care system would be decided by following how many insurances and how many diseases, and the process time is very sensible to the record size, because if the size is big, the processing time would be increased. Then the system surely gets stress. Therefore, the partial process such as encryption/security level is a necessity. Security management, as is suggested, consists of u#.profile to involve the patient's information such as name, sex, address, i#.info which is insurance information, and d#.info that lists all disease information in a server. In case the patient calls, as soon as the sensor at home detects the patient, it registers the patient information automatically from the call to the hospital and shares its information with the doctor. Then the security management in the server decides its security level, key, policies of license, and security. With this scenario, all systems would be used remotely in a network on the cloud. In the suggested system, we have set security functions like an encryption and authentication in the system; however, each step has threats like our analysis results showed. The patient, medical clinic, and server can be attacked by an attacker by repudiation, tampering, spoofing, DDoS, information disclosure, eavesdropping/forgery modulation attack to medical record, and exposure of personal information by medical information sharing. To protect against these threats, we define the security management with a license policy, security policy, and key management with the DRM server and DRM agent and we summarized the security strategies. We expect these strategies to help when we set the real health care system on the cloud in the future.

## Figures and Tables

**Algorithm 1:**
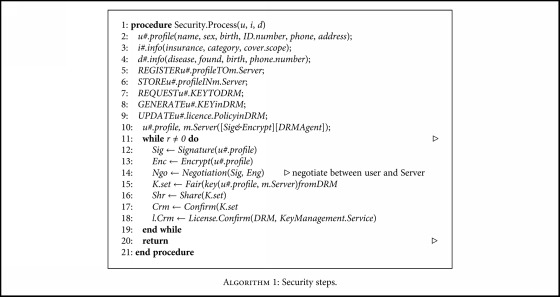
Security steps.

**Table 1:** Damage records.

Date	Damage
February 14, 2013, Froedtert Hosp, USA	Acquiring privilege by inserting a malicious code into an employee's PC, leaking about 43,000 patients' personal information such as patient personal insurance certificate, card information, and social security number.

Barnaby Jack, 2012, RSA Conference, USA	The hacker approaches the patient using an insulin pump and exploits the vulnerability of the small computer inside the insulin pump.

Korea, 2013	(i) Collecting various medical information from a domestic hospital using an overseas server. (ii) Medical records, prescription lists, and MRI images; not only hospital medical information but also the sales status of pharmaceutical companies. (iii) The hacker does not stop leaking medical information and takes control of the PC inside the hospital. (iv) Many medical institution PCs are infected with a malicious code. (v) Many hackers can remotely administer hospital PCs to arbitrarily manipulate prescriptions.

**Table 2:** Threats to medical contents.

Threats	Contents
Repudiation	(i) Cannot receive data from a source outside the trust boundary. Consider using logging or auditing to record the source, time, and summary. (ii) Device claims: cannot write data received from an entity on the other side of the trust boundary.

Tampering	(i) Subject to a persistent cross-site scripting attack because it does not sanitize data storage “device” inputs/outputs and to cross-site scripting attacks. (ii) Reading or modifying data transmitted over an authenticated dataflow. (iii) Tampering by an attacker and leading to corruption of device. (iv) Attack via log files.

Spoofing	(i) Be spoofed by an attacker, leading to information disclosure. Consider using a standard authentication mechanism to identify the destination process. (ii) Be spoofed by an attacker, leading to incorrect data delivered to web server. (iii) Be spoofed by an attacker, leading to data being written to the attacker's target instead of the device.

DDoS	(i) A DDoS attack to a server, which connects to a user device, a biosensor, will be a potential threat that makes a service impossible. (ii) Resource consumption can be hard to deal with, and there are times that it makes sense to let the OS do the job.

Information disclosure	(i) Data flowing across generic dataflow may be sniffed by an attacker. It can be used to attack other parts of the system or simply be a disclosure of information leading to compliance violations. (ii) When u#.profile, i#.info, and d#.info are required to be shared for patient movement, they have to share them with weak security.

Eavesdropping/forgery	(i) Attack to personal information and medical records which transfers between a biosensor and a server, a medical system and a server, or a user device and a server.

**Table 3:** Requirement to lightweight process.

Items	Contents
Code size	Because a sensor is a small device and has a limitation, the algorithm should have a small size for it to run, achieved by reducing the number of code line.
Security strength	To process, the structure will be compacted; however, the encryption strength has to be kept strong.
Fast speed	The code should be optimized to speed up by decreasing the number of code lines and by removing useless codes.
Low energy consumption	To use IoT devices, a sensor usually uses an encryption algorithm, but the devices which are used in a home health care system have a limitation of having a small size, so low energy consumption is necessary, which may be achieved by decreasing the number of rounds in the encryption.
